# Obesity Surgery Mortality Risk Score as a Predictor for Intensive Care Unit Admission in Patients Undergoing Laparoscopic Bariatric Surgery

**DOI:** 10.3390/jcm13082252

**Published:** 2024-04-12

**Authors:** Paola Aceto, Roberto De Cicco, Claudia Calabrese, Irene Marusco, Filippo Del Tedesco, Ersilia Luca, Cristina Modesti, Teresa Sacco, Liliana Sollazzi, Luigi Ciccoritti, Francesco Greco, Piero Giustacchini, Francesco Pennestrì, Pierpaolo Gallucci, Marco Raffaelli

**Affiliations:** 1 Department of Emergency, Anesthesiologic and Reanimation Sciences, Fondazione Policlinico Universitario Agostino Gemelli IRCCS, 00168 Rome, Italy; roberto.decicco@policlinicogemelli.it (R.D.C.); claudia.calabrese@policlinicogemelli.it (C.C.); irene.marusco@policlinicogemelli.it (I.M.); filippo.deltedesco@policlinicogemelli.it (F.D.T.); cristina.modesti@policlinicogemelli.it (C.M.); teresa.sacco@policlinicogemelli.it (T.S.); liliana.sollazzi@unicatt.it (L.S.); 2 Department of Basic Biotechnological Science, Intensive and Peri-Operative Clinics, Università Cattolica del Sacro Cuore, 00168 Rome, Italy; 3 Division of Endocrine and Metabolic Surgery, Fondazione Policlinico Universitario Agostino Gemelli IRCCS, 00168 Rome, Italy; luigi.ciccoritti@policlinicogemelli.it (L.C.); francesco.greco@policlinicogemelli.it (F.G.); piero.giustacchini@policlinicogemelli.it (P.G.); francesco.pennestri@policlinicogemelli.it (F.P.); pierpaolo.gallucci@policlinicogemelli.it (P.G.); marco.raffaelli@unicatt.it (M.R.); 4 Centro di Ricerca di Chirurgia delle Ghiandole Endocrine e dell’Obesità, Università Cattolica del Sacro Cuore, 00168 Rome, Italy

**Keywords:** obesity, bariatric surgery, anaesthesia, intensive care unit, postoperative care

## Abstract

**Background:** Laparoscopic bariatric surgery provides many benefits including lower postoperative pain scores, reduced opioid consumption, shorter hospital stays, and improved quality of recovery. However, the anaesthetic management of obese patients requires caution in determining postoperative risk and in planning adequate postoperative pathways. Currently, there are no specific indications for intensive care unit (ICU) admission in this surgical population and most decisions are made on a case-by-case basis. The aim of this study is to investigate whether Obesity Surgery Mortality Risk Score (OS-MRS) is able to predict ICU admission in patients undergoing laparoscopic bariatric surgery (LBS). **Methods**: We retrospectively reviewed data of patients who underwent LBS during a 2-year period (2017–2019). The collected data included demographics, comorbidities and surgery-related variables. Postoperative ICU admission was decided via bariatric anaesthesiologists’ evaluations, based on the high risk of postoperative cardiac or respiratory complications. Anaesthesia protocol was standardized. Logistic regression was used for statistical analysis. **Results:** ICU admission was required in 2% (*n* = 15) of the 763 patients. The intermediate risk group of the OS-MRS was detected in 84% of patients, while the American Society of Anaesthesiologists class III was reported in 80% of patients. A greater OS-MRS (*p* = 0.01), advanced age (*p* = 0.04), male gender (*p* = 0.001), longer duration of surgery (*p* = 0.0001), increased number of patient comorbidities (*p* = 0.002), and previous abdominal surgeries (*p* = 0.003) were predictive factors for ICU admission. **Conclusions:** ICU admission in obese patients undergoing LBS is predicted by OS-MRS together with age, male gender, number of comorbidities, previous abdominal surgeries, and duration of surgery.

## 1. Introduction

Within the context of a considerable increase in pathologies related to obesity, bariatric surgery is often the only solution to the problem, as it reduces the long-term incidence of cardiovascular events and improves survival [1,2]. In this context, the laparoscopic approach offers many benefits, including reduced use of opioids, a shorter hospital stay, and faster postoperative recovery. However, the anaesthetic management of the obese patient is complex, since it requires an accurate assessment in order to estimate the risk of complications and consequently plan adequate postoperative treatment [3,4]. Even with often conflicting results, hospitalization in a postoperative intensive care unit (ICU) after bariatric surgery is mainly associated with risk factors such as advanced age; BMI greater than 50 kg/m^2^; male gender; some specific comorbidities, such as OSAS and ischemic heart disease; duration of surgery; and intraoperative complications [5,6]. The Obesity Surgery Mortality Risk Score (OS-MRS) system is a validated scale to predict postoperative risk which classifies obese patients into high, intermediate, or low risk, depending on age, body mass index, sex, and other comorbidities such as hypertension and history of pulmonary embolism [7]. The OS-MRS [8] system has never previously been investigated in terms of establishing the need for hospitalization in an intensive care unit. If at the beginning of bariatric surgery intensive monitoring was considered almost as a routine, today the percentage of these hospitalizations has drastically reduced, currently reaching an average of 5% [5]. Aside from the use of laparoscopic surgery, the introduction of opioid-sparing analgesia and the reversal of neuromuscular blockades are the key points that have made this reduction possible. The latest 2019 guidelines developed by authoritative American scientific societies (AACE/TOS/ASMBS/OMA/ASA 2019) recommend the monitoring in intensive care of patients with high cardiological and respiratory risks, without, however, establishing specific predictive factors for ICU admission [9,10]. Even today, without clear indications, decisions are made by the anaesthesiologist based on subjective assessments [11,12].

This study aims to investigate whether the Obesity Surgery Mortality Risk Score (OS-MRS) system is able to predict ICU admission in patients undergoing laparoscopic bariatric surgery (LBS).

## 2. Materials and Methods

### 2.1. Study Design and Population

The medical records of patients undergoing LBS between 1 August 2017 and 31 December 2019 were analysed at Fondazione Policlinico Universitario A. Gemelli IRCCS (Rome). All patients with class III (pathological obesity with BMI > 40 kg/m^2^) or class II obesity (severe obesity characterized by a BMI between 35.00 and 39.99 kg/m^2^) were considered. Exclusion criteria were previous bariatric surgery and an incomplete data report.

### 2.2. Anaesthetic Protocol

The anaesthetic protocol, which was standardized, included preoperative evaluation and intraoperative management. During the preoperative anaesthesiologic visit made by a dedicated team during pre-hospitalization, all demographic and anamnestic parameters (comorbidities) were recorded. The OS-MRS was also calculated, by assigning one point to each of the following five preoperative variables: age ≥ 45 years, male gender, BMI ≥ 50 kg/m^2^, arterial hypertension, and the presence of risk factors for pulmonary thromboembolism (previous thromboembolism, preoperative vena cava filter, hypoventilation, and pulmonary hypertension). The class of the risk assigned to each patient was dependent on the total points obtained. Therefore, three risk classes were established: low risk, or class A (score of zero or one point); moderate risk, or class B (two or three points); and high risk, or class C (four or five points). 

On the day of surgery, the patients were placed on the operating table in an anti-Trendelenburg position with an inclination of 30 degrees and in the so-called “ramped position”, which allows the alignment of the oral–pharyngeal, pharyngeal–laryngeal and laryngo–tracheal axes and the horizontal alignment of the external auditory meatus and jugule, in order to compensate for the exaggerated flexion of the head caused by the presence of dorsal (hump) and cervical fat, thus facilitating manual ventilation and tracheal intubation manoeuvres [13]. This position was maintained for the entire surgery, until the patient was extubated. Intraoperative vital sign monitoring included an electrocardiogram; non-invasive blood pressure monitoring; oximetry; end-tidal CO_2_ partial pressure (EtCO_2_); end-tidal volatile anaesthetic concentration; body temperature, measured by oesophageal probe; and mioresolution by cinemiograph (E-NMT module, GE Healthcare, Chicago, IL, USA). The depth of anaesthesia was monitored using BIS (Bispectral Index, Covidien, Mansfield, MA, USA). Before the induction of anaesthesia, all patients underwent pre-oxygenation using a face mask with FiO_2_ = 1 for 3–5 min. Anaesthesia was induced by administering a bolus of propofol at a dosage of 2.5 mg/kg (IBW), fentanyl 3 mcg/kg (IBW), and rocuronium bromide 1.2 mg/kg (IBW). The ideal body weight (IBW) was calculated using the Broca formula: height (cm)—100 in men and height (cm)—104 in women. Orotracheal intubation was performed upon reaching a TOF = zero with a Macintosh blade n. 5 in men and n. 4 in women. In case of non-intubation after two attempts with conventional laryngoscopy, the Glidescope was used as an alternative device. Volume-controlled protective mechanical ventilation was performed by setting a tidal volume of 6 mL/kg (IBW), a respiratory rate of 12–16 breaths per minute in order to maintain EtCO_2_ values between 35 and 40 mmHg, and a positive end-expiratory pressure (PEEP) of 5–12 mmHg. The standardized anaesthesia protocol was followed, with sevoflurane at Bispectral Index (BIS)-guided concentrations in order to ensure a value below 60, with fentanyl up to the maximum dosage of 5 mcg/kg (IBW), and with continuous infusion of remifentanil, whilst maintaining the blood pressure and heart rate values within 20% of the baseline [14]. Rocuronium was administered at a dosage of 1.2 mg/kg of ideal body weight (IBW) with additional doses (boluses of 0.15 mg/kg IBW) to induce and maintain a deep NMB (TOF = 0 and PTC ≤ 2) until the end of the surgical procedure (at the end of the entero-enteric anastomosis in the gastric bypass, gastro-enteric in the mini bypass, and up to the end of the gastric resection in the sleeve gastrectomy), guided by neuromuscular monitoring. The intra-abdominal pressure values induced with pneumoperitoneum were maintained between 12 and 14 mmHg throughout the procedure [15,16]. Once the surgical wound was sutured, the residual curarization was antagonized with sugammadex according to the neuromuscular monitoring guide (NMT): 2 mg/kg (total body weight—TBW) in the presence of two responses to the TOF stimulation (moderate block), or 4 mg/kg (TBW) in the presence of a TOF = 0 and a PTC ≥ 2 (deep block). Sevoflurane was discontinued upon reaching a TOF ratio of ≥ 0.7. Bariatric surgery was performed by the same surgical team.

After awakening, patients were monitored in the Post Anaesthesia Care Unit or were transferred to the Intensive Care Unit (ICU). Postoperative ICU admission was planned by the bariatric anaesthetist based on the following criteria:

1. High risk of postoperative cardiac complications requiring close and prolonged hemodynamic monitoring.
2. High risk of postoperative respiratory complications requiring mechanical ventilation.

### 2.3. Statistical Analysis

Data are presented as mean and standard deviation (or median with interquartile range) for continuous variables and as numbers (percentages) for dichotomous or discrete variables. Student’s *t*-test and the chi-squared test were used to analyse continuous and dichotomous or discrete variables, respectively. In order to investigate the contributions of possible predictive factors for postoperative ICU admission (dependent variable), the independent variables examined were age, gender, BMI, number and type of comorbidities, preoperative diagnosis of OSAS, ASA class, Obesity Surgery Mortality Risk Score (OS-MRS), and duration of surgery. A logistic regression analysis was performed, including only the significant variables in the univariate analysis, and the results were reported as odds ratio (OR) with a 95% confidence interval. The cut-off for the most clinically relevant variables was also calculated by choosing the variable with the specificity > 90% and, in case of equal specificity, the one with the highest sensitivity. Data with a value of *p* < 0.05 were considered statistically significant. The software used for the statistical analysis was STATA 14.

## 3. Results

**Figure 1:**
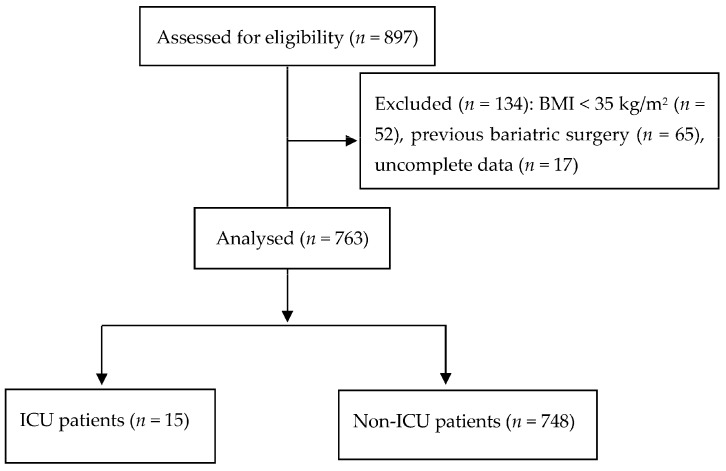
Flow chart. Abbreviations: BMI, body mass index; ICU, intensive care unit.

In our study, we analysed 763 patients (Figure 1). The intermediate risk group of the OS-MRS system was detected in 84% of patients, while the American Society of Anaesthesiologists’ class III was reported in 80% of patients. Postoperative admission to the ICU was necessary for 15 patients (2%). Patients admitted to a postoperative ICU had a higher average age (50.1 years ± 10.8 vs. 43.8 ± 10.2 years for non-ICU patients, t = −2.91; *p* = 0.038). Gender was another statistically significant variable; of the 15 ICU patients, 13 were male (232/748 in the non-ICU group, χ² = 6.69; *p* = 0.001). Finally, a higher BMI was found in the ICU patients—48.9 ± 10.3 kg/m^2^ vs. 44.7 ± 6.5 kg/m^2^ for ICU and non-ICU patients, respectively (t = −2.94; *p* = 0.034). The patients’ comorbidities are reported in Table 1.

The OS-MRS, number of comorbidities, duration of the surgery, and previous number of abdominal interventions were significantly associated with ICU admission (see Table 2 and Table 3).

During the multivariate analysis (see Table 3), a greater OS-MRS (*p* = 0.01); advanced age (*p* = 0.04); male gender (*p* = 0.001); longer duration of surgery (*p* = 0.0001); increased number of the patient’s comorbidities (*p* = 0.002); and previous abdominal surgeries (*p* = 0.003) were predictive factors for ICU admission.

Early postoperative complications (within 30 days) occurred in 15 patients of the non-ICU group.

There was no difference in the rate of 30-day postoperative complications (6.7% vs. 2.0%, χ² = 1.43; *p* = 0.23) for ICU versus non-ICU patients. Regarding the type of complications, one patient in the ICU group showed hemoperitoneum. In the non-ICU group, there were 15 cases of intraoperative complications, including anastomotic leak (*n* = 1); stenosis or hernias with obstruction (*n* = 2); anaemia (*n* =5); wound infection (*n* =1); anastomosis or endoluminal bleeding (*n* = 5); and cardiac complication (*n* = 1). Postoperative complications based on the different types of bariatric surgery and outcomes (Clavien–Dindo classification) are shown in Table 4. Regarding the Clavien–Dindo classification, seven patients (0.92% of the total sample) in the non-ICU group showed grade II, whereas eight patients (1.05% of the total sample) showed grade IIIb (one in the ICU group and seven in the non-ICU group).

## 4. Discussion

The main finding of this study was that a high OS-MRS was able to predict ICU admission in patients undergoing laparoscopic bariatric surgery. Furthermore, this study showed differences in anthropometric parameters and comorbidities in both the ICU and non-ICU groups, indicating how these variables can support the decision to admit or not admit a patient to the ICU after laparoscopic bariatric surgery. In particular, factors not included in the OS-MRS, such as the number of comorbidities and the surgery duration, should be considered when planning ICU admission. Not all factors underlying OS-MRSs require ICU admission since, for example, BMI alone was not a significant predictive factor in the multivariate analysis. The advantage of the OS-MRS is the combination of some factors that can be life-threatening if rated together when performing the score. On the other hand, factors such as surgical expertise, hospital operation volume, and the multidisciplinary team’s level of support play crucial roles in determining the need for ICU admission, which OS-MRSs alone may not fully capture. Therefore, solely relying on OS-MRSs to predict ICU admission may be insufficient, and further studies need to consider additional factors to ensure comprehensive patient care and safety.

Our results are in line with the current literature regarding male gender, older age, and duration of surgery as independent predictors of ICU admission [11,17,18,19,20]. The male gender is more frequently characterized by android-type obesity, defined by a WHR > 0.85 in women and >0.90 in men. The latter is associated with an increase in perioperative complications, especially affecting the respiratory system, which often requires monitoring in the ICU. The prevalence of android obesity in males could explain the presence of generally more altered gas exchange in men than in women [21]. In this regard, it has been shown that WHR is more predictive than BMI for postoperative respiratory changes. In our study, a higher BMI was found in ICU patients. However, BMI was not an independent predictor of ICU admission. Therefore, according to our data, we can affirm that it is not correct to plan admission to the ICU exclusively based on BMI. This finding is in contrast with the practice of many hospitals, which, especially in the past, have considered a BMI > 50 kg/m^2^ as an independent predictor of postoperative admission to the ICU. Today, it is possible to say that BMI certainly represents a risk factor for postoperative complications, but its effects on mortality are questionable as well as its use as a predictor of ICU admission. Indeed, two important meta-analyses [5,22] have shown that a higher BMI decreases mortality in obese patients admitted to ICU: “the obesity paradox” [23]. Our study showed age as an independent predictor of ICU admission with a cut-off of ≥ 58 years. This result is in line with the current literature and can be explained by the fact that advancing age is associated with the persistence of obesity and worse morbid conditions linked to it. The study made by the Australian group Morgan, conducted on a large sample (12,062 patients undergoing laparoscopic bariatric surgery), supports our result, demonstrating that age is a significant predictor of ICU admission [24]. The same is true for another important study conducted on 828 elderly patients undergoing sleeve gastrectomy. The percentage of ICU admission was higher (24%) than in other studies and largely justified by advanced age [11]. The number and type of comorbidities clearly represent a further point of discussion. The complexity of the postoperative management of obese patients is related to the number of diseases that are often associated with obesity. In our study, we also found that the number of comorbidities is an independent predictor of ICU admission, and that patients admitted to ICU had five or more comorbidities in most cases. The OSAS preoperative diagnosis is a much-discussed criterion in the literature. If, according to some studies, it may represent a predictor of ICU admission, recent evidence seems to deem superfluous the monitoring in an intensive setting after bariatric surgery of patients regularly on CPAP treatment [25]. This category of patients has been shown to be at low risk of cardiorespiratory complications after bariatric surgery [26,27,28,29]. Surgery duration was more often associated with postoperative admission to the ICU. Most studies that have analysed morbidity and mortality attributable to bariatric surgeries [30,31] suggest that the latter are related to the complexity of the procedure, the number of operations performed by the centre, and the surgical technique (best outcome in the laparoscopic technique). The surgeon’s experience can also influence the duration of surgery and, consequently, postoperative admission to the ICU [32,33]. In our study, unlike the study of Froylich et al. [34], postoperative admission to the ICU after bariatric surgery was not associated with a significant increase in postoperative complications at 30 days, and no respiratory failure occurred in any of the 15 patients admitted to the ICU in our study. Our study has some limitations. First of all, it is a retrospective and monocentric study. Since retrospective analysis depends on the review of charts that were originally not designed to collect data for research, some information is bound to be missing. For this reason, we excluded medical records with missing data. Second, the surgeries were performed by a single team. On the one hand, standardization of surgical procedures allowed us to reduce the bias related to the surgeon’s experience, likely enhancing patient safety and outcomes; on the other hand, the results are poorly applicable on a large scale. Therefore, relevant variables not assessed in this study, such as surgical expertise and hospital operation volume, should be considered in future multicentric studies. Third, the study did not foresee external validation on a larger series that could lead to the integration of the risk factors into routine clinical practice to support clinician decisions for ICU admission.

Moreover, our study had a relatively short follow-up period; thus, it could have underestimated the potential benefits of ICU admission. An extended follow-up could have provided important information about outcomes. In this regard, this study did not include patients undergoing bariatric surgery after 2019 specifically to avoid bias due to a different utilization of critical care services during the COVID-19 pandemic. Acknowledging these limitations is crucial for interpreting the study findings accurately and guiding future research efforts to address these constraints and further enhance understanding of the topic.

The strength of this study is represented by the standardized anaesthesia protocol, since strict monitoring of neuromuscular block during anaesthesia and appropriate reversing at the end of surgery are both essential in the setting of bariatric surgery. The advantage of this approach was to maintain a constant deep neuromuscular blockade (NMB), improving surgical conditions during laparoscopic bariatric surgery as well as increasing surgeon satisfaction with the workspace [16]. The antagonization of neuromuscular block at the end of surgery based on the monitoring of muscular function offers a unique strategy to avoid postoperative recurarization and the associated respiratory complications almost frequent in this population. In this clinical context, it is necessary to remember that deep neuromuscular block can be only antagonized by sugammadex which facilitates a rapid and complete recovery, minimizes the risk of postoperative curarization, and allows bariatric fast-track surgery [35]. As sugammadex needs to be dosed according to block depth, neuromuscular monitoring is mandatory in this setting [35]. Another advantage of neuromuscular monitoring was the chance to discontinue sevoflurane anaesthesia based on the train-of-four ratio, ensuring a smooth transition from anaesthesia to the recovery phase. This protocol helps in the timely emergence of the patient from anaesthesia, optimizing safety whilst preventing the risk of muscle paralysis perception.

In this regard, the anaesthesia depth was also standardized. We used BIS to guide anaesthetic administration, which is an index derived from the analysis of the electroencephalogram spectrum, i.e., brainwave patterns recorded from electrodes placed on the patient’s forehead. BIS-guided anaesthesia is particularly useful in the setting of bariatric surgery in order to avoid light anaesthesia while minimizing the risk of excessive sedation that could impair the quality of postoperative recovery and impact subsequent complications [14].

Using ideal body weight rather than actual weight for determining the dosage of anaesthetic agents during surgery is a common practice in the treatment of obese patients. It has been demonstrated that using the actual weight of obese individuals may lead to overdose of anaesthetic agents, with subsequent potential complications such as prolonged emergence from anaesthesia, respiratory depression, and cardiovascular instability. Obese patients have altered pharmacokinetics and pharmacodynamics due to changes in their volume of distribution, tissue perfusion, and metabolism. Therefore, by dosing anaesthesia based on IBWs of morbidly obese patients, anaesthesia providers aim to achieve a balance between providing adequate anaesthesia for the surgical procedure and minimizing the risk of adverse effects [36]. Overall, this study also suggests an innovative approach, possible thanks to the advances in modern anaesthesia relating to multimodal pain management and the complete and safe reversal of neuromuscular blockades, which allow a faster recovery of the patient suffering from severe obesity.

The novelty of this study is represented by the chance to calculate the likelihood of admission to the ICU at the time of anaesthesiologic visit, thus, before patients’ hospitalization. Obesity Surgery Mortality Risk Scores could effectively stratify obese patients scheduled for laparoscopic bariatric surgery into groups with either high or low risk of postoperative ICU admission, offering clinical value in the personalized selection and direct management of resources. This could help both anaesthesiologists and surgeons in improving operating room planning and surgical case scheduling, which in turn play a critical role in the efficient matching of supply and demand for surgery. In this regard, future studies could investigate the potential power of OS-MRSs in improving patient selection for surgical schedules, and thus, operating room workflow. In our opinion, this study adds valuable insights to the body of knowledge about risk factors for ICU admission and contributes to the ongoing efforts to optimize perioperative care and patient outcomes in bariatric surgery.

## 5. Conclusions

In our study, only 2% of patients undergoing laparoscopic bariatric surgery required ICU admission. The OS-MRS predicted ICU admission, as did greater age, male gender, number of comorbidities, and duration of surgery. On the contrary, BMI was not a reliable parameter to indicate the need for monitoring in an intensive environment. By basing the indications of ICU admission on the severity of obesity rather than on a dimensional parameter such as BMI, we achieved the double advantage of favouring a faster turnover of the operating room and reducing inappropriate admissions to the ICU, with a significant impact on costs.

## Figures and Tables

**Table 1:** Comorbidities of ICU patients and non-ICU patients.

Comorbidity (Yes/No)	ICU (*n* = 15)	NON-ICU (*n* = 748)	χ²	*p*
OSAS	9/6	213/535	7.08	0.008
Bronchial Asthma	4/11	66/682	5.62	0.02
Arterial Hypertension	12/3	312/436	8.82	0.003
Ischemic Heart Disease	3/12	13/735	23.89	0.0001
Diabetes Mellitus II	7/8	177/571	4.25	0.04
Dyslipidaemia	9/6	153/595	13.75	0.0001
Dysthyroidism	1/14	179/569	2.43	0.12
GRD	10/5	183/565	13.86	0.0001
Anaemia	3/13	22/726	5.21	0.02
Liver Disease	10/5	165/583	16.55	0.0001
Osteoarthritis	1/14	28/720	0.34	0.56
DVT	0/15	12/736	0.24	0.62
Pulmonary Embolism	0/15	2/746	0.04	0.84
OS-MRS (0-1-2-3)	0-1-13-1	78-323-302-42	13.64	0.003
ASA Score (II-III)	3/12	151/597	0.0003	0.99

Abbreviations: ICU, intensive care unit; OSAS, obstructive sleep apnea syndrome; ASA, American Society of Anaesthesiologists; OS-MRS, Obesity Surgery Mortality Risk Score; GRD, gastroesophageal reflux disease; DVT, deep vein thrombosis.

**Table 2:** Surgery characteristics of ICU patients and non-ICU patients.

	ICU (*n* = 15)	NON-ICU (*n* = 748)	χ²	*p*
Type of Surgery (1-2-3-4) *	3-9-2-1	148-461-107-32	0.21	0.976
Duration of Surgery	171.1 ± 80.5	68.4 ± 32.8	−11.48	0.0001
Previous abdominal Surgery **	0/1/2/6/5/1	29/364/299/42/2/8	214.33	0.0001

* Sleeve gastrectomy/gastric bypass/mini gastric bypass/SADI or SADI-S. ** Number of patients who underwent previous abdominal surgery (from 0 to 5). ICU, intensive care unit.

**Table 3:** Multivariate regression.

Variable	OR	95%CI	SE	Z	*p*
OS-MRS	0.02	0.001–0.43	0.03	−2.51	0.01
Surgery duration	1.04	1.02–1.07	0.12	3.68	0.0001
Previous abdominal surgery	3.76	1.55–9.15	1.71	2.92	0.003
Age	1.16	1.01–1.34	0.08	2.07	0.04
Gender	0.004	0.001–0.09	0.006	−3.36	0.001
BMI	1.03	0.92–1.15	0.06	0.54	0.59
Number of comorbidities	3.03	1.49–6.16	1.10	3.07	0.002

OR, odds ratio; OS-MRS, Obesity Surgery Mortality Risk Score; BMI, body mass index; 95%CI, 95% confidence interval; SE, standard error.

**Table 4:** Types of postoperative complications in ICU vs. non-ICU patients.

	ICU (*n* = 15)	NON-ICU (*n* = 748)
Postoperative complication, *n* (type of bariatric surgery)		
Anastomotic leak	0	1 (MGBP)
Stenosis or hernias with intestinal obstruction	0	2 (GBP)
Anaemia	0	5 (2 S; 2 GBP; 1 MGBP)
Hemoperitoneum	1 (GBP)	0
Wound infection	0	1 (S)
Anastomosis or endoluminal bleeding	0	5 (1 MGBP; 4 GBP)
Cardiac complications *	0	1 (GBP)
Outcome **, *n* (type of bariatric surgery)		
II	0	7 (3 S; 3 GBP; 1 MGBP)
IIIb	1 (GBP)	8 (2 MGBP; 6 GBP)

ICU, Intensive Care Unit; MGBP, mini gastric bypass; GBP, gastric bypass; S, sleeve gastrectomy; * atrial fibrillation; ** based on Clavien–Dindo classification.

## Data Availability

The data presented in this study are available upon request.

## References

[1] Arterburn D.E., Telem D.A., Kushner R.F., Courcoulas A.P. (2020). Benefits and Risks of Bariatric Surgery in Adults. JAMA.

[2] WHO (2000). Obesity: Preventing and Managing the Global Epidemic. Report of a WHO Consultation. World Health Organ. Tech. Rep. Ser..

[3] Riley C.L. (2022). Anesthesia and Enhanced Recovery After Surgery in Bariatric Surgery. Anesthesiol. Clin..

[4] Buchwald H., Avidor Y., Braunwald E., Jensen M.D., Pories W., Fahrbach K., Schoelles K. (2004). Bariatric Surgery. JAMA.

[5] Pompilio C.E., Pelosi P., Castro M.G. (2016). The Bariatric Patient in the Intensive Care Unit: Pitfalls and Management. Curr. Atheroscler. Rep..

[6] Steinbrook R. (2004). Surgery for Severe Obesity. N. Engl. J. Med..

[7] Younus H., Chakravartty S., Sarma D.R., Patel A.G. (2018). Endobarrier as a Pre Bariatric Surgical Intervention in High-Risk Patients: A Feasibility Study. Obes. Surg..

[8] García-García M.L., Martín-Lorenzo J.G., Lirón-Ruiz R., Torralba-Martínez J.A., García-López J.A., Aguayo-Albasini J.L. (2017). Failure of the Obesity Surgery Mortality Risk Score (OS-MRS) to Predict Postoperative Complications After Bariatric Surgery. A Single-Center Series and Systematic Review. Obes. Surg..

[9] Mechanick J.I., Apovian C., Brethauer S., Garvey W.T., Joffe A.M., Kim J., Kushner R.F., Lindquist R., Pessah-Pollack R., Seger J. (2019). Clinical Practice Guidelines For The Perioperative Nutrition, Metabolic, and Nonsurgical Support of Patients Undergoing Bariatric Procedures—2019 Update: Cosponsored By American Association of Clinical Endocrinologists/American College of Endocrinology, The Obesity Society, American Society For Metabolic & Bariatric Surgery, Obesity Medicine Association, and American Society of Anesthesiologists. Endocr. Pract..

[10] WHO (2008). Waist Circumference and Waist-Hip Ratio: Report of a WHO Expert Consultation.

[11] Khidir N., EL-Matbouly M., Al Kuwari M., Gagner M., Bashah M. (2018). Incidence, Indications, and Predictive Factors for ICU Admission in Elderly, High-Risk Patients Undergoing Laparoscopic Sleeve Gastrectomy. Obes. Surg..

[12] Robert M., Pasquer A., Espalieu P., Laville M., Gouillat C., Disse E. (2014). Gastric Bypass for Obesity in the Elderly: Is It as Appropriate as for Young and Middle-Aged Populations?. Obes. Surg..

[13] Aceto P., Perilli V., Modesti C., Ciocchetti P., Vitale F., Sollazzi L. (2013). Airway Management in Obese Patients. Surg. Obes. Relat. Dis..

[14] Aceto P., Lai C., Perilli V., Sacco T., Modesti C., Raffaelli M., Sollazzi L. (2016). Factors Affecting Acute Pain Perception and Analgesics Consumption in Patients Undergoing Bariatric Surgery. Physiol. Behav..

[15] Aceto P., Perilli V., Modesti C., Sacco T., De Cicco R., Ceaichisciuc I., Sollazzi L. (2020). Effects of Deep Neuromuscular Block on Surgical Workspace Conditions in Laparoscopic Bariatric Surgery: A Systematic Review and Meta-Analysis of Randomized Controlled Trials. Minerva Anestesiol..

[16] Aceto P., Modesti C., Sacco T., De Cicco R., Perilli V., Raffaelli M., Lai C., Sollazzi L. (2018). Patient-Related Factors Predicting Workspace Conditions during Laparoscopic Bariatric Surgery. Obes. Surg..

[17] Van Den Broek R.J.C., Buise M.P., Van Dielen F.M., Bindels A.J.G.H., Van Zundert A.A.J., Smulders J.F. (2009). Characteristics and Outcome of Patients Admitted to the ICU Following Bariatric Surgery. Obes. Surg..

[18] DeMaria E.J., Portenier D., Wolfe L. (2007). Obesity Surgery Mortality Risk Score: Proposal for a Clinically Useful Score to Predict Mortality Risk in Patients Undergoing Gastric Bypass. Surg. Obes. Relat. Dis..

[19] DeMaria E.J., Murr M., Byrne T.K., Blackstone R., Grant J.P., Budak A., Wolfe L. (2007). Validation of the Obesity Surgery Mortality Risk Score in a Multicenter Study Proves It Stratifies Mortality Risk in Patients Undergoing Gastric Bypass for Morbid Obesity. Ann. Surg..

[20] Alanzi A., Alamannaei F., Abduljawad S., Ghuloom A., Alahmed F.A., Alzaidani A.E., Almusaifer M.F., Alanezi M.A., Adeel S. (2023). Patient Outcomes and Rate of Intensive Care Unit Admissions Following Bariatric Surgery: A Retrospective Cohort Study of 775 Patients. Cureus.

[21] Zavorsky G.S., Hoffman S.L. (2008). Pulmonary Gas Exchange in the Morbidly Obese. Obes. Rev..

[22] Anzueto A., Frutos-Vivar F., Esteban A., Bensalami N., Marks D., Raymondos K., Apezteguía C., Arabi Y., Hurtado J., González M. (2011). Influence of Body Mass Index on Outcome of the Mechanically Ventilated Patients. Thorax.

[23] Fernandez A.Z., Demaria E.J., Tichansky D.S., Kellum J.M., Wolfe L.G., Meador J., Sugerman H.J., MacDonald K.G., Hawkins M.L., O’Leary J.P. (2004). Multivariate Analysis of Risk Factors for Death Following Gastric Bypass for Treatment of Morbid Obesity. Ann. Surg..

[24] Morgan D.J.R., Ho K.M. (2016). The Anaesthetic Assessment, Management and Risk Factors of Bariatric Surgical Patients Requiring Postoperative Intensive Care Support: A State-Wide, Five-Year Cohort Study. Anaesth. Intensive Care.

[25] De Jong A., Chanques G., Jaber S. (2017). Mechanical Ventilation in Obese ICU Patients: From Intubation to Extubation. Crit. Care.

[26] DeMaria E.J., Carmody B.J. (2005). Perioperative Management of Special Populations: Obesity. Surg. Clin. N. Am..

[27] Shearer E., Magee C.J., Lacasia C., Raw D., Kerrigan D. (2013). Obstructive Sleep Apnea Can Be Safely Managed in a Level 2 Critical Care Setting after Laparoscopic Bariatric Surgery. Surg. Obes. Relat. Dis..

[28] Grover B.T., Priem D.M., Mathiason M.A., Kallies K.J., Thompson G.P., Kothari S.N. (2010). Intensive Care Unit Stay Not Required for Patients with Obstructive Sleep Apnea after Laparoscopic Roux-En-Y Gastric Bypass. Surg. Obes. Relat. Dis..

[29] Goucham A.B., Coblijn U.K., Hart-Sweet H.B., de Vries N., Lagarde S.M., van Wagensveld B.A. (2016). Routine Postoperative Monitoring after Bariatric Surgery in Morbidly Obese Patients with Severe Obstructive Sleep Apnea: ICU Admission Is Not Necessary. Obes. Surg..

[30] Sakran N., Sherf-Dagan S., Blumenfeld O., Romano-Zelekha O., Raziel A., Keren D., Raz I., Hershko D., Gralnek I.M., Shohat T. (2018). Incidence and Risk Factors for Mortality Following Bariatric Surgery: A Nationwide Registry Study. Obes. Surg..

[31] Goulenok C., Monchi M., Chiche J.D., Mira J.P., Dhainaut J.F., Cariou A. (2004). Influence of Overweight on ICU Mortality: A Prospective Study. Chest.

[32] Søvik T.T., Aasheim E.T., Kristinsson J., Schou C.F., Diep L.M., Nesbakken A., Mala T. (2009). Establishing Laparoscopic Roux-En-y Gastric Bypass: Perioperative Outcome and Characteristics of the Learning Curve. Obes. Surg..

[33] Di Lorenzo N., Furbetta F., Favretti F., Segato G., De Luca M., Micheletto G., Zappa M., De Meis P., Lattuada E., Paganelli M. (2010). Laparoscopic Adjustable Gastric Banding via Pars Flaccida versus Perigastric Positioning: Technique, Complications, and Results in 2549 Patients. Surg. Endosc..

[34] Froylich D., Corcelles R., Davis M., Boules M., Daigle C.R., Schauer P.R., Brethauer S.A. (2016). Factors Associated with Length of Stay in Intensive Care after Bariatric Surgery. Surg. Obes. Relat. Dis..

[35] Carron M., Veronese S., Foletto M., Ori C. (2013). Sugammadex Allows Fast-Track Bariatric Surgery. Obes. Surg..

[36] Petrini F., Di Giacinto I., Cataldo R., Esposito C., Pavoni V., Donato P., Trolio A., Merli G., Sorbello M., Pelosi P. (2016). Perioperative and Periprocedural Airway Management and Respiratory Safety for the Obese Patient: 2016 SIAARTI Consensus. Minerva Anestesiol..

